# Evaluation of Osteonecrosis Risk Using Serum C-Terminal Cross-Linked Telopeptide of Type 1 Collagen (CTX-1) Levels in Osteoporotic Patients: Effects of Drug Holidays and Risk Factors

**DOI:** 10.5152/eurasianjmed.2026.251104

**Published:** 2026-01-15

**Authors:** Emine Esra Ergül, Oğuzhan Laçin, Hamide Özge Kılıçaslan, Fahrettin Bostancı, Elif Can Özdemir, Ayşegül Kılıç, Serhat Hayme, Hilal Büşra Ayçiçek

**Affiliations:** 1 Department of Physical Medicine and Rehabilitation, Gülhane Training and Research Hospital, Ankara, Türkiye; 2 Department of Biostatistics, Erzincan Binali Yıldırım University Faculty of Medicine, Erzincan, Türkiye

**Keywords:** Bisphosphonate, CTX-1, osteonecrosis

## Abstract

**Background:**

The aim of this study is to evaluate the risk of osteonecrosis of the jaw in patients receiving osteoporosis treatment using C-terminal cross-linked telopeptide of type 1 collagen (CTX-1) values, to analyze follow-ups after drug holidays, and to compare treatment agents and other risk factors to determine the association with CTX-1 results.

**Methods:**

A total of 273 patients (266 female and 7 male) who received bisphosphonate and denosumab treatment for osteoporosis were included in this retrospective study. Sociodemographic characteristics, vitamin D levels, serum CTX-1 level, presence of diseases affecting CTX-1 levels, type of bisphosphonate, duration of use, presence of drug holidays, and duration of denosumab use (if any) were recorded. The effects of bisphosphonates and denosumab on CTX-1 levels were compared, and differences in the risk of osteonecrosis between them were evaluated.

**Results:**

In this study, a meaningful association was not identified between serum CTX-1 levels and the measured vitamin D values (*P* = .232). Patients who underwent a drug holiday had significantly higher mean serum CTX-1 levels (266.2 ± 175.1 pg/mL) compared to those without a drug holiday (199.9 ± 138.5 pg/mL; *P* = .009). Higher CTX-1 levels were observed in individuals receiving ibandronate and alendronate, whereas the lowest values were detected in patients treated with denosumab.

**Conclusion:**

Serum CTX-1 levels appeared unaffected by vitamin D. Although the denosumab group exhibited the highest risk of osteonecrosis, the difference compared to zoledronic acid was not statistically significant. These results suggest caution during jaw-related procedures and consideration of drug holidays when necessary.

**Main Points**

- No meaningful association was observed between circulating vitamin D levels and serum C-terminal cross-linked telopeptide of type 1 collagen (CTX-1) concentrations. This suggests that CTX-1 levels may be a more direct marker than vitamin D in assessing the risk of osteonecrosis.
- Patients who had a drug holiday showed significantly higher CTX-1 levels. These findings support the potential benefit of drug holidays in reducing suppression of bone turnover before dental procedures.
- C-terminal cross-linked telopeptide of type 1 collagen levels were lowest in patients treated with denosumab, who also had the highest risk of jaw osteonecrosis. However, the osteonecrosis risk in denosumab users was not significantly different from that in patients treated with zoledronic acid.

## Introduction

The World Health Organization defines osteoporosis as a progressive, systemic skeletal disorder marked by reduced bone mass and microarchitectural deterioration, leading to increased bone fragility and a heightened fracture risk. With rising life expectancy, osteoporosis has become a major public health concern. In Türkiye, prevalence among individuals ≥50 years is estimated at 22.2% in men and 27.2% in women.^1^

Bisphosphonate-related osteonecrosis of the jaw (BRONJ) is an infrequent but clinically significant complication of bisphosphonate therapy, which is commonly utilized in the management of osteoporosis and in the prevention of skeletal complications due to bone metastases.^2^ The condition was first formally described in 2003 by Marx et al,^3^ highlighting a critical adverse effect linked to long-term bisphosphonate use. However, it was found that not only these groups of medications cause jaw osteonecrosis but also monoclonal antibodies against RANKL (Receptor Activator of Nuclear Factor Kappa-B Ligand) can increase the risk of osteonecrosis.^4^ Medication-related osteonecrosis of the jaw (MRONJ), previously recognized as BRONJ, should be evaluated primarily based on the underlying therapeutic indication—whether for malignancy or osteoporosis/osteopenia. The risk is considerably elevated in oncology patients, with estimates approaching but generally remaining below 5%, whereas individuals treated for osteoporosis exhibit a much lower incidence, typically less than 0.05%.^5^ Antiresorptive agents such as bisphosphonates and denosumab primarily function by reducing bone turnover. Although these 2 drug classes operate via different biological pathways, both lead to decreased bone remodeling and are associated with MRONJ. This suggests a potential etiological link between impaired bone remodeling and the onset of MRONJ.^5^^,^^6^ This hypothesis is further reinforced by the role of osteoclast activity at sites of bone injury, as well as the observed increase in MRONJ risk with prolonged treatment duration and higher cumulative doses.^5^^,^^7^

Bone-turnover markers offer a quantitative assessment of bone remodeling activity. Various markers have been investigated for their potential predictive relevance and clinical applicability in individuals receiving antiresorptive treatment. Among these are C-terminal telopeptide of type 1 collagen (CTX-1), osteocalcin, N-terminal telopeptide, and alkaline phosphatase. Of these, fasting morning serum CTX-1 is one of the most frequently utilized indicators in clinical practice.^8^ Marx et al^3^ suggested a risk stratification model based on serum CTX-1 levels, categorizing values below 100 pg/mL as high risk, between 100 and 150 pg/mL as moderate risk, and above 150 pg/mL as low risk. Nonetheless, current literature presents conflicting views on the clinical utility of this marker. While some studies support the reliability of CTX-1 as an indicator of bone turnover,^3^^,^^9^ others caution against its routine use for this purpose due to insufficient and inconsistent evidence.^10^^-^^12^

The American Academy of Oral Medicine has concluded that CTX-1 testing is not suitable for the clinical evaluation and management of patients receiving antiresorptive treatment, due to a lack of robust evidence supporting its diagnostic accuracy and the absence of well-defined sensitivity and specificity parameters.^8^ Consequently, this study did not aim to promote CTX-1 for routine clinical application, but instead focused on exploring its potential role in assessing MRONJ risk among patients receiving antiresorptive agents who are scheduled to undergo minor or major dentoalveolar procedures. Additionally, the aim was to determine whether there are differences in CTX-1 levels between different treatment modalities and to examine a possible association between serum CTX-1 concentrations and vitamin D levels.

## Material and Methods

This retrospective study included patients who underwent serum CTX-1 testing prior to dental procedures due to antiresorptive drug use, as recorded in the physical medicine and rehabilitation outpatient clinic between January 1, 2023, and December 31, 2024. According to Lazarovici et al,^10^ the required minimum sample size was estimated at 88, considering an effect size of 0.3, statistical power of 80%, and a significance threshold of 0.05. This calculation was conducted using G*Power version 3.1 (Heinrich Heine University, Düsseldorf, Germany). In total, 273 participants (266 women and 7 men) were included in the study. The study population consisted of 273 individuals, 266 of whom were female and 7 male. The presentation of this research adhered to the principles outlined in the STROBE (Strengthening the Reporting of Observational Studies in Epidemiology) statement.

Age, gender, vitamin D levels, vitamin D ranges, diabetes mellitus, hypertension, rheumatologic diseases, and comorbidities potentially affecting CTX-1 levels were recorded. Antiresorptive drug use was classified and recorded as alendronate, ibandronate, risedronate, zoledronic acid, and denosumab. In addition, the presence and duration of drug holiday and previous bisphosphonate history of patients using denosumab were also determined.

C-terminal cross-linked telopeptide of type 1 collagen values were used in the analyses both directly and categorized according to risk groups. In accordance with previous reports, serum CTX-1 levels were used to categorize the risk of osteonecrosis as follows: levels between 300 and 600 pg/mL were considered to indicate no risk; 150-299 pg/mL suggested none to minimal risk; 101-149 pg/mL represented a moderate risk; and levels below 100 pg/mL were classified as high risk. These thresholds were applied to stratify patients in the current study.^13^

This study received ethical approval from the ethics committee of Non-Interventional Clinical Research Ethics Committee of University of Health Sciences, Gülhane Training and Research Hospital on May 8, 2025 (Decision No: 2025/71) and was carried out in full compliance with the Declaration of Helsinki guidelines. Written informed consent was secured from all individuals after they were briefed on the nature and aims of the study.

### Statistical Analysis

All statistical analyses were performed using IBM SPSS Statistics version v. 25.0 (IBM SPSS Corp.; Armonk, NY, USA). The distribution of data was evaluated for normality through the Shapiro–Wilk test, and Levene’s test was applied to verify the equality of variances. Descriptive statistics were presented as mean ± SD when variables followed a normal distribution, and as median with minimum and maximum values for non-normally distributed data. The Mann–Whitney *U*-test was employed for comparing 2 independent groups based on median values, while group comparisons involving 3 or more categories were analyzed using the Kruskal–Wallis test. Relationships between categorical variables were examined using Pearson’s chi-square test, and associations between continuous variables were assessed via Spearman’s rank correlation. A *P*-value less than .05 was interpreted as statistically significant.

## Results

Demographic characteristics and comorbidities are summarized in Table 1. Regarding vitamin D status, 72 individuals (26.4%) had deficiency (<20 ng/mL), 74 (27.1%) had insufficiency (21-29 ng/mL), and 126 (46.2%) had sufficient levels (>30 ng/mL). Additionally, 1 patient exhibited vitamin D toxicity (>150 ng/mL). Overall, 76.9% of the participants had received vitamin D supplementation.

Descriptive statistics for age, vitamin D status, and drug holiday duration across the CTX-1–based osteonecrosis risk groups are presented in Table 2. No significant correlation was found between serum CTX-1 levels and age (*r* = −0.039, *P* = .520). Similarly, there was no significant association between numerical vitamin D levels and CTX-1 concentrations (*r* = 0.073, *P* = .232). When vitamin D was analyzed categorically as deficiency, insufficiency, sufficiency, and toxicity, no significant correlation was identified with CTX-1 (*r* = 0.074, *P* = .222). Furthermore, there was no significant difference in CTX-1 levels between the presence and absence of vitamin D intake (*P* = .234). Comorbidities such as rheumatologic diseases, diabetes mellitus, and hypertension were also not associated with CTX-1 concentrations (*P* = .138, *P* = .864, and *P* = .416, respectively).

Patients’ antiresorptive medications were categorized as alendronate, ibandronate, risedronate, zoledronic acid, or denosumab, and their corresponding serum CTX-1 levels are presented in Table 3. Serum CTX-1 concentrations varied significantly according to the type of antiresorptive agent used (*P* < .001). Higher CTX-1 levels were observed in individuals receiving ibandronate and alendronate, whereas the lowest levels were recorded in those treated with denosumab, reflecting its potent inhibitory effect on bone turnover. Pairwise comparisons confirmed that the differences between denosumab and both alendronate and ibandronate were statistically significant (*P* = .004 and *P* < .001, respectively). However, the outcomes observed with denosumab and zoledronic acid were similar, with no meaningful differences detected between the 2 treatments (*P* = .309).

Among the 85 patients who were receiving denosumab both prior to and at the time of evaluation, significantly lower mean serum CTX-1 levels were observed (180.8 ± 143.4 pg/mL) compared to those not on denosumab (229.3 ± 150.6 pg/mL; *P* = .002), suggesting a more pronounced suppression of bone turnover associated with ongoing denosumab use. Patients who received bisphosphonates prior to denosumab exhibited significantly lower mean CTX-1 levels (172.1 ± 134.7 pg/mL) compared to those without prior bisphosphonate exposure (238.8 ± 160.3 pg/mL; *P* = .002), indicating a cumulative suppressive effect on bone turnover.

Patients who underwent a drug holiday had significantly higher mean serum CTX-1 levels (266.2 ± 175.1 pg/mL) compared to those without a drug holiday (199.9 ± 138.5 pg/mL; *P* = .009), indicating increased bone turnover during treatment interruption. There was a low positive correlation between drug holiday duration and serum CTX-1 levels (*r* = 0.385, *P *= .002), indicating that longer treatment interruptions may be associated with increased bone turnover.

## Discussion

This study has 3 main findings. Firstly, the analysis revealed no statistically meaningful link between quantitative vitamin D measurements and CTX-1 concentrations. Secondly, whether participants received vitamin D supplements or not had no notable impact on CTX-1 concentrations. Thirdly, serum CTX-1 concentrations varied significantly according to the type of antiresorptive agent used.

This study recruited 274 women and men, randomly selected from Turkish population with the aim of exploring the potential association between serum CTX-1 concentrations and vitamin D levels. Although vitamin D is essential for skeletal health, its variability within routine clinical ranges does not appear to track real-time resorptive activity captured by CTX-1. Markers of bone turnover demonstrate significant variability across different ethnic populations. Because bone-remodeling markers (BRMs) exhibit ethnic and geographic variability owing to genetics, diet, sunlight exposure, cultural dress, and healthcare access, findings from non-Turkish cohorts cannot be assumed to generalize.^14^ Numerous studies on this topic have been reported in the literature with different ethnic populations. So far, no research has specifically examined the potential relationship between serum CTX-1 concentrations and vitamin D levels within the Turkish population. This is the first study to investigate this topic in Turkish population.

Results from studies conducted in various ethnic groups are not consistent. Multiple population-based studies show that the association observed between vitamin D status and the bone-resorption marker CTX varies markedly by geography and ethnicity:

- Japan: CTX rises sharply in the 50s and stays high in older age; vitamin D_3_ is generally higher after menopause but dips in the early postmenopausal phase.
- Norway: Despite widespread vitamin D_3_ insufficiency/deficiency, serum CTX shows no association with vitamin D and averages lower than in German and present pre-menopausal cohorts.
- India: Median CTX is similar in pre- and postmenopausal women, peaking in the sixth decade; even with >60% vitamin D deficiency, CTX does not differ by vitamin D status.
- Italy: Higher vitamin D levels were associated with lower CTX-1 values in serum, suggesting an inverse correlation.^15^

Overall, these data highlight substantial inter-ethnic and geographic variation in CTX levels and their relationship with vitamin D. These findings align with previous research, showing no detectable relationship between serum CTX-1 values and vitamin D levels among individuals in the Turkish population. Moreover, it was proposed that the widespread, long-standing vitamin D deficiency seen in Turkish populations triggered an adaptive skeletal response in which BRMs, after rising transiently, gradually returned to baseline as the deficiency persisted.

This lack of correlation suggests that within a typical outpatient range, vitamin D status may function primarily as a permissive factor for mineralization rather than a linear driver of bone resorption. Null effects could also reflect range restriction (few patients with severe deficiency), seasonal and assay variability, or unmeasured confounding (e.g., renal function, inflammation).^16^

Thiering et al^17^ showed that bone-turnover markers exhibited clear seasonality and CTX reflects bone resorption and follows a circadian rhythm. Because blood-draw time and season were not standardized in this cohort—factors that materially influence CTX due to its diurnal and seasonal variability—any underlying relationship with 25(OH)D may have been biased toward the null, resulting in the observed lack of correlation.^17^

Variability in circulating vitamin D across cohorts may partly explain the wide differences reported in BRM levels. Vitamin D deficiency predisposes individuals to secondary hyperparathyroidism, a state that should, in theory, heighten skeletal turnover and thus elevate BRMs. Yet, the literature does not consistently corroborate a link between vitamin D status and BRM concentrations: numerous studies have found no relationship between serum BRMs and either 25-hydroxyvitamin D or parathyroid hormone.^18^

Although vitamin D is widely used for the prevention and treatment of musculoskeletal problems, studies on its impact on bone mineral density and fracture risk have yielded diverse and sometimes conflicting results. Differences in treatment outcomes may stem from various factors, including the type, amount, and length of vitamin D intake, as well as individuals’ initial 25(OH)D levels, all of which can shape how the body responds to supplementation.^19^

Moreover, even though vitamin D supplementation raises the levels of serum 25(OH)D in individuals with deficiency, it does not reliably produce a corresponding reduction in serum CTX levels. There are different randomized controlled trials showing mixed and limited evidence that vitamin D supplementation alters bone-turnover markers.^20^ Andersen et al^21^ found that although vitamin D3 supplementation (administered at daily doses of 10 and 20 mg) successfully raised serum vitamin D concentrations, it had no measurable impact on BRMs.

Consistent with these findings, MacDonald et al^22^ also observed that bone turnover indicators such as procollagen type 1 N-terminal propeptide (P1NP) and s-CTX did not differ meaningfully between the groups analyzed. They proposed that measured serum 25(OH)D concentrations might not reliably indicate either true physiological vitamin D stores or the clinical effectiveness of supplementation.^22^

A placebo-controlled clinical trial employing a double-blind randomized design explored how vitamin D supplementation influenced bone turnover biomarkers. A group of 197 participants with 25(OH)D levels <30 ng/mL (mean age 60.2 ± 11.1 years; 47% female) were divided into 2 groups and supplied either 2800 IU of vitamin D daily or a placebo for 8 weeks. The results revealed that indicated that supplementation with vitamin D did not significantly influence serum concentrations of either CTX or P1NP. Within individuals demonstrating reduced 25(OH)D concentrations, no meaningful changes in bone-turnover markers were detected after 8 weeks of vitamin D treatment.^23^

In this study, postmenopausal women and elderly men with osteoporosis who were receiving antiresorptive treatment were investigated. This analysis indicated that CTX-1 concentrations remained largely unchanged regardless of whether participants received vitamin D supplementation. At the center, evaluating vitamin D status and correcting any deficiencies before beginning antiresorptive treatment is regarded as a critical step to ensure both the effectiveness and safety of therapy. As a result, none of the participants had markedly low serum vitamin D levels, and all were classified as having adequate vitamin D status. Consequently, no significant variation in CTX-1 concentrations was found between those who received supplementation and those who did not.

It is noteworthy that this study demonstrated no observable correlation between serum vitamin D levels and CTX-1 concentrations. Although vitamin D deficiency is implicated in impaired bone remodeling and mucosal healing, its influence on biochemical markers of bone resorption such as CTX-1 appears limited. Nevertheless, considering the extensively documented importance of vitamin D in maintaining skeletal integrity, ensuring sufficiency prior to antiresorptive therapy remains a cornerstone of MRONJ prevention strategies.

### Type of Antiresorptive Agent and Drug Holiday

The purpose of this research was to investigate how serum CTX-1 concentrations relate to different clinical factors in individuals undergoing antiresorptive treatment before dental interventions. These findings revealed that serum CTX-1 levels significantly differed according to the type of antiresorptive agent, with denosumab users displaying the lowest levels, indicative of potent suppression of bone turnover. These are consistent with prior studies reporting denosumab’s strong inhibitory effect on osteoclastic activity via RANKL blockade.^5^^,^^7^

The pathogenesis of MRONJ remains unclear. Evidence suggests multiple contributing factors: bone remodeling, including initial infection and inflammation, soft tissue cytotoxicity, angiogenesis inhibition, and degenerative changes following exposure to bisphosphonates or denosumab.^24^

As denosumab-treated MRONJ emerges, it is becoming increasingly clear that osteoclast dysfunction underlies its pathophysiology.^5^ Denosumab in menopausal patients with osteoporosis/osteopenia was associated with an increase in CD14⁺/CD11b⁺ osteoclast precursors (OCPs) after a subsequent dose, despite persistent suppression of bone-turnover markers (CTX, PINP, TRACP [tartrate-resistant acid phosphatase]). This discrepancy suggests that RANKL blockade may inhibit precursor-to-osteoclast differentiation, thus allowing studies of OCPs while resorptive activity remains biochemically quiescent.^25^

According to a study in cancer patients by Limones et al,^29^ denosumab use is associated with a significantly higher risk of MRONJ compared with zoledronic acid. It cannot be currently determine whether this effect is due to the drug itself or to the duration of treatment and/or follow-up.^26^

Our data also showed that prior bisphosphonate exposure and the absence of drug holidays were associated with lower CTX-1 levels, supporting the notion that bone turnover is cumulatively suppressed by sequential antiresorptive therapies. This is consistent with previous reports that long-term or concomitant use of bisphosphonates and denosumab increases the risk of MRONJ due to sustained inhibition of bone remodeling.^7^^,^^27^

Interestingly, higher CTX-1 levels were observed in patients undergoing drug holidays, suggesting a partial improvement in bone turnover. However, the clinical benefit of drug holidays remains controversial. Some studies, such as those included in the American Association of Oral and Maxillofacial Surgeons (AAOMS) 2022 position paper, suggest potential benefits in select cases,^5^ while others highlight the lack of robust evidence linking drug holidays to a reduced incidence of MRONJ.^28^ Furthermore, Hallmer et al^28^ suggested that periodontitis and dental infections, rather than bone-turnover markers alone, may have a more pronounced effect.

Consequently, clinicians are advised to adopt preventive strategies such as comprehensive oral examinations with appropriate radiographs, oral hygiene education, maintenance of good oral health, and completion of indicated dental treatment before initiating antiresorptive therapy. It was also noted that implant placement or removal—or even the presence of implants—may trigger MRONJ in patients receiving high-dose antiresorptive therapy.^29^

Surgical treatment strategies in the context of established MRONJ also deserve special mention. These findings support the need for comprehensive risk assessment, as surgical treatment, particularly when performed early and with appropriate debridement techniques, achieves high success rates, as demonstrated in prospective studies.^28^^,^^30^

In their systematic review and meta-analysis, Ghio et al^31^ concluded that CTX is not a reliable predictor of MRONJ risk in cancer patients and has low predictive value in osteoporosis populations.

The pathogenesis of MRONJ is complex and cannot be explained solely by suppression of bone remodeling. In this context, risk estimation based on serum CTX is inherently challenging, and recent reports suggest restricting its use for this purpose.

This study has limitations, including its retrospective design and the potential for confounding by variables inherent to this type of analysis. Furthermore, these findings support the limited predictive value of CTX-1 and highlight the need for multifactorial risk assessment models that integrate clinical, radiographic, and laboratory data. Emerging clinical prediction tools leveraging extensive pharmacovigilance databases may provide more accurate MRONJ risk stratification in the future.

This study corroborates the limited utility of serum CTX-1 levels in isolation as a predictor of MRONJ risk. Instead, attention should be focused on comprehensive preoperative assessments, optimization of modifiable risk factors—including vitamin D status—and meticulous dental management to minimize the occurrence of this serious complication.

## Figures and Tables

**Table 1.** Demographic Characteristics of the Cases

	**n = 273**
Age (years)*	62.90 ± 9.53
Gender, n (%) Female Male	266 (97.4) 23 (51.1)
Diabetes mellitus, n (%) No Yes	227 (83.2) 46 (16.8)
Hypertension, n (%) No Yes	167 (61.2) 106 (38.8)
Rheumatolohic disease, n (%) No Yes	247 (90.5) 26 (9.5)

*Descriptive statistics are shown as mean ± SD.

**Table 2.** Descriptive Statistics of Clinical Variables by C-Terminal Cross-Linked Telopeptide of Type 1 Collagen Risk Groups

**CTX-1 Risk ** **Group**	**n (%)**	**Age ** **(mean ± SD)**	**Vitamin D ** **(mean ± SD)**	**Drug Holiday ** **(mean ± SD)**	**CTX-1 ** **(mean ± SD)**
No risk	61 (22.3)	60.26 ± 9.78	32.37 ± 16.79	2.79 ± 1.95	439.18 ± 117.32
Moderate risk	107 (39.2)	64.24 ± 9.31	30.63 ± 13.93	2.12 ± 2.08	216.36 ± 43.88
Moderate-to-high risk	32 (11.7)	64.50 ± 11.72	28.88 ± 12.31	1.50 ± 0.87	117.50 ± 13.20
High risk	73 (26.7)	62.45 ± 8.18	31.41 ± 18.69	1.11 ± 0.53	65.01 ± 22.68
**Total**	273	62.90 ± 9.53	31.03 ± 15.78	2.12 ± 1.83	214.09 ± 149.56

CTX-1, C-terminal cross-linked telopeptide of type 1 collagen.

**Table 3.** Distribution of Serum C-Terminal Cross-Linked Telopeptide of Type 1 Collagen Concentrations Among Antiresorptive Agents

**Antiresorptive Agents**	**Median (pg/mL)**	**Minimum**	**Maximum**	**Mean ± SD (pg/mL)**	**N**
Alendronate	240.00	25.00	700.00	241.00 ± 153.96	65
Risedronate	205.00	50.00	700.00	218.57 ± 164.31	14
Ibandronate	230.00	30.00	650.00	263.59 ± 139.46	39
Zoledronic acid	165.00	25.00	620.00	205.28 ± 142.08	88
Denosumab	120.00	6.00	670.00	163.06 ± 134.12	64
Total	185.00	6.00	700.00	212.99 ± 146.99	270
